# The comprehensive expression analysis of circular RNAs in gastric cancer and its association with field cancerization

**DOI:** 10.1038/s41598-017-15061-w

**Published:** 2017-11-06

**Authors:** Amanda Ferreira Vidal, André M. Ribeiro-dos-Santos, Tatiana Vinasco-Sandoval, Leandro Magalhães, Pablo Pinto, Ana K. M. Anaissi, Samia Demachki, Paulo Pimentel de Assumpção, Sidney Emanuel Batista dos Santos, Ândrea Ribeiro-dos-Santos

**Affiliations:** 1 Laboratory of Human and Medical Genetics, Federal University of Pará, Belém, Pará, Brazil; 2 Center of Oncology Research, Federal University of Pará, Belém, Pará, Brazil

## Abstract

Circular RNAs comprise a new class of long noncoding RNAs characterized by their 5′ and 3′ ends covalently joined. Previous studies have demonstrated that some circular RNAs act as microRNA sponges, and are associated with cellular proliferation in cancer. We were the first to analyze the global expression of circular RNAs in samples of patients without gastric cancer, gastric cancer, and matched tumor-adjacent gastric tissue. Among the samples, we identified 736 previously annotated circular RNAs by RNA-Seq. The tumor-adjacent tissue presented the higher abundance of circular RNAs and could not be considered as a normal tissue, reinforcing the notion of field effect in gastric cancer. We identified five differentially expressed circular RNAs that may be potential biomarkers of this type of cancer. We also predicted candidate microRNAs targets of the highest expressed circular RNAs in gastric tissues and found five miRNAs. Overall, our results support the hypothesis of circular RNAs representing a novel factor in the dynamic epigenetic network of gene regulation, which involves the microRNAs, its mRNAs targets, and the circular RNAs-derived genes. Further studies are needed to elucidate the roles and the functional relevance of the circular RNAs in human diseases.

## Introduction

Circular RNAs comprise a new class of long noncoding RNAs characterized by their 5′ and 3′ ends covalently joined. They were misinterpreted as splicing errors for more than 20 years until their rediscovery in 2012 as diverse, highly abundant, conserved and naturally occurring RNAs in eukaryotes^1–5^.

About 90,000 different circular RNAs were described in human, which most are derived mainly from annotated exons (~85%) and a smaller fraction from untranslated regions (UTRs), introns and unannotated regions of the genome. They are most commonly formed from two or three exons, comprising between a hundred and four thousand nucleotides in length^1,2,5–7^.

These RNA molecules are likely generated by a process known as back-splicing. This noncanonical splicing can produce three types of circular RNAs, in which they are classified: exonic circular RNAs (circRNAs), circular intronic RNAs (ciRNAs) and exon-intron circular RNAs (EIciRNAs)^1,8–11^. CircRNAs are predominantly cytoplasmic and were reported acting as microRNAs (miRNAs) and RNA-binding proteins (RBPs) sponges. CiRNAs and EIciRNAs are enriched in the nucleus and are RNA polymerase II-associated, suggesting that they promote the transcription of their parent genes^2,5,8,12,13^.

Circular RNAs molecules are easily accessed and measured in body fluids and have distinct characteristics such as tissue-specificity and stability in both intra and extracellular environments. This suggest their potential as clinical markers that may provide new insights into the prevention and treatment of several diseases^14^.

Although neither their biogenesis nor roles have been entirely understood, circular RNA expression has already been described as altered in human diseases such diabetes, atherosclerosis, Alzheimer’s disease and cancer^15–17^. On cancer, they were associated with cellular proliferation, and some clinical features such as tumor size and presence of distal metastases^14,18–21^.

Among different cancers, gastric cancer remains the third leading cause of cancer-related death worldwide. Due the lack of specific symptoms, most gastric cancer patients are diagnosed in advanced-stage disease with a poor prognosis^22^. Some reports have shown that recurrence of gastric cancer may be due the field cancerization (or field effect) in gastric mucosa. According to this theory, the tissue surrounding tumors, despite being histologically normal, shares molecular abnormalities that are present in fully developed tumors^23–25^. Multiple genetic and epigenetic alterations, mostly DNA methylation and miRNA abnormal expression, have been described as field effect biomarkers in gastric cancer, reinforcing the occurrence of a field effect in this type of cancer^26,27^.

MiRNAs are a class of small nonconding RNAs involved in many biological processes by blocking target mRNAs translation^28^. The epigenetic network in which the miRNAs participate is complex and dynamic since its involves not only target mRNAs, but also other types of noncoding RNAs such as the circular RNAs^14^. Given that some circular RNAs act as miRNAs sponges, they may also have a potential epigenetic regulation role in gastric cancer.

The aim of this study was to identify, characterize and compare the entirety of all expressed circular RNAs in samples of patients without gastric cancer, gastric cancer samples and matched tumor-adjacent gastric tissue. Additionally, we correlated circular RNAs’ expression data with miRNA expression.

## Results

We performed RNA-Seq on ribosomal-depleted total RNA isolated from gastric tissue samples. Head-to-tail back-spliced junctions were detected by using two combined prediction algorithms (Supplementary Fig. 1).

**Figure 1:**
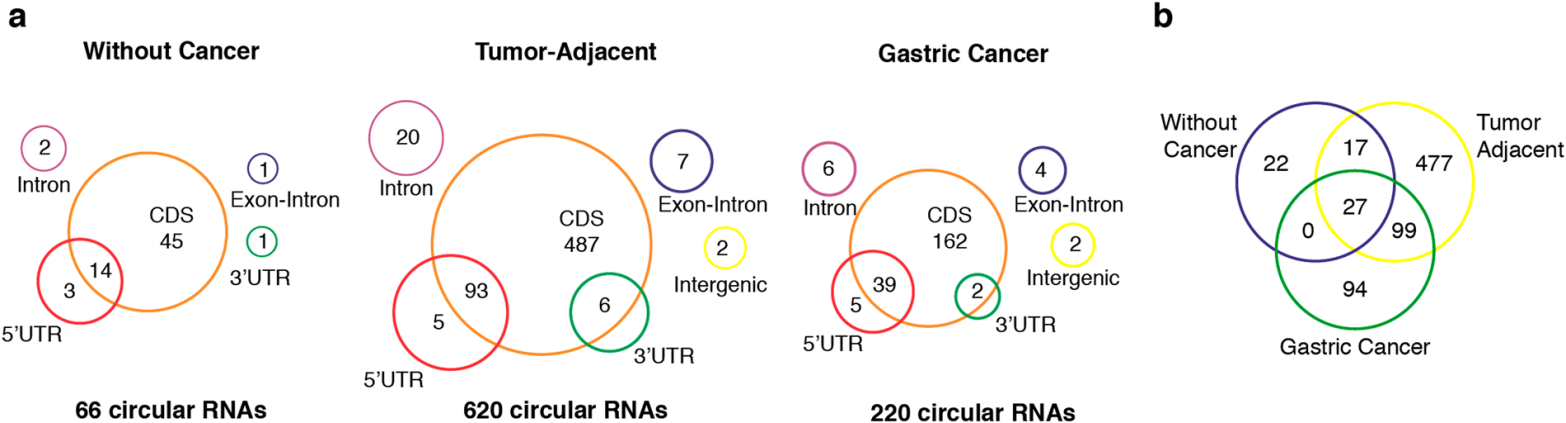
Total of annotated circular RNAs detected in gastric tissue. (**a**) Number of expressed circular RNAs in each type of gastric tissue according to their origin. (**b**) Venn diagram of all expressed circular RNAs between the three types of gastric tissue. CDS: coding DNA sequence.

In total, we detected 736 unique annotated circular RNAs in all three groups of gastric tissues. As shown in Fig. 1a, we identified 66 annotated circular RNAs in gastric tissue without gastric cancer, 620 in matched tumor-adjacent gastric tissue and 220 in gastric cancer samples.

A previous study showed that most of human circular RNAs contain two or three exons^29^. To further evaluate this data, we analyzed the number of exons per circular RNA in gastric tissue and found similar results (Supplementary Fig. 2). As shown in Table 1, the number of exons is not necessarily related to the circular RNAs spliced lengths. A notable example is that hsa_circ_0004176, which harbors 26,767 nt in length, spans only two exons, while hsa_circ_0020397, which harbors 2,738 nt in length, spans 26 exons.

**Table 1:** Transcript features of the expressed circular RNAs in gastric tissues.

Attribute/Type of gastric tissue	Without cancer	Tumor-adjacent	Gastric cancer
Longest circular RNA	*hsa_circ_0000246 (*MCU*)	*hsa_circ_0000230 (*ZEB1*)	hsa_circ_0004176 (*IFT43*)
	7620 nt-3 exons	88220 nt-4 exons	26767 nt-2 exons
Shortest circular RNA	hsa_circ_0055734 (*ANKRD36*)	hsa_circ_0000439 (*ATXNK2*)	hsa_circ_0000439 (*ATXNK2*)
	98 nt spliced	97 nt	97 nt
Circular RNA with the highest number of exons	hsa_circ_0020397 (*DOCK1*)	hsa_circ_0001613 (*SENP6*)	hsa_circ_0023923 (*PICALM*)
	26 exons-2738 nt	12 exons-1722 nt	11 exons-1128 nt
Gene with the highest number of expressed circular RNA isoforms	*XPO1*	*UBAP2*	*UBAP2*
	hsa_circ_0001017 (307 nt-3 exons)	hsa_circ_0001849 (119 nt -2 exons)	hsa_circ_0001849 (119 nt -2 exons)
	hsa_circ_0001016 (2 exons)	hsa_circ_0001851 (159 nt-2 exons)	hsa_circ_0001851 (159 nt-2 exons)
	*UBAP2*	hsa_circ_0001847 (377 nt-4 exons)	hsa_circ_0001847 (377 nt-4 exons)
	hsa_circ_0001849 (119 nt -2 exons)	hsa_circ_0005993 (187 nt-3 exons)	hsa_circ_0001850 (278 nt-4 exons)
	hsa_circ_0001851 (159 nt 2 exons)		

*Exon-intron circular RNA.

Interestingly, *UBAP2* gene presented five different circular RNA isoforms expressed in gastric tissue, suggesting that circular alternative splicing is also occurring in the stomach (Table 1).

To examine the genomic localization of gastric circular RNAs, we analyzed the number of circular RNAs per chromosome, and found that most of them is derived from chromosome 1 of the human genome (Supplementary Fig. 3).

Although most gastric circular RNAs had less than 10 back-spliced junction reads of coverage, some highly expressed circular RNAs in matched adjacent gastric tissue had a read count of more than 35. Table 2 shows the most expressed circular RNAs in gastric tissue without gastric cancer, matched tumor-adjacent gastric tissue and gastric cancer samples.

**Table 2:** List of the most expressed circular RNAs in gastric tissue.

Gastric tissue	Circular RNA	Number of back spliced junction reads	Gene symbol
Without cancer	hsa_circ_0001340	6	*TMCC1*
	hsa_circ_0006354	5	*VAMP3*
	hsa_circ_0001380	3	*UBXN7*
	hsa_circ_0000419	3	*RAB3IP*
	hsa_circ_0002496	3	*APPBP2*
	hsa_circ_0004368	3	*REPS1*
	hsa_circ_0001112	3	*DGKD*
Tumor-adjacent	hsa_circ_0001092*	41	*CFLAR*
	hsa_circ_0000284*	37	*HIPK3*
	hsa_circ_0000437*	37	*CORO1C*
	hsa_circ_0001136*	31	*ASXL1*
	hsa_circ_0001445	28	*SMARCA5*
	hsa_circ_0000211*	26	*SFMBT2*
	hsa_circ_0001498	21	*WDR41*
Gastric cancer	hsa_circ_0000437*	17	*CORO1C*
	hsa_circ_0001136*	15	*ASXL1*
	hsa_circ_0000211*	14	*SFMBT2*
	hsa_circ_0000284*	13	*HIPK3*
	hsa_circ_0001092*	11	*CFLAR*
	hsa_circ_0001897	11	*POMT1*
	hsa_circ_0001727	8	*ZKSCAN1*

*Circular RNAs in common between tumor-adjacent and gastric cancer tissues.

**Figure 2:**
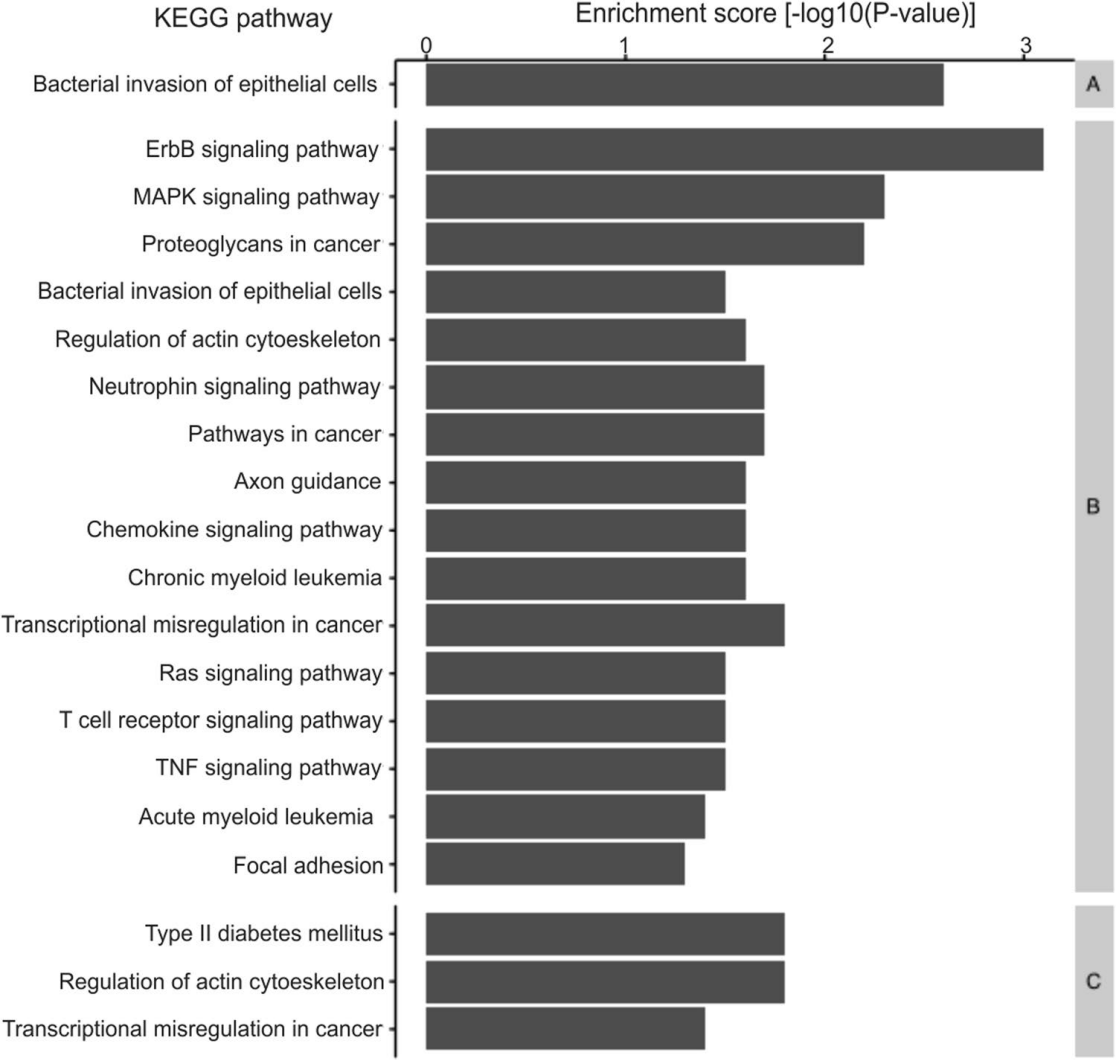
GO enrichment of the gastric circular RNAs-derived genes, evidencing the KEGG pathways and its scores. (**A**) Gastric tissue without gastric cancer. (**B**) Matched tumor-adjacent gastric tissue. (**C**) Gastric cancer.

To further explore the potential function of the expressed circular RNAs in gastric tissue, we selected the gastric circular RNAs-derived genes to perform GO enrichment analysis (Fig. 2). The gastric tissue without gastric cancer and matched tumor-adjacent gastric tissue circular RNAs-derived genes were enriched in the process of bacterial invasion of epithelial cells, such as *Salmonella* sp., *Listeria* sp. and *Shigella* sp^30^ (Supplementary Fig. 4). Tumor-adjacent gastric tissue circular RNAs-derived genes also were enriched in cancer-related processes, as well as gastric cancer’s.

Circular RNAs can regulate miRNAs by sequestering them by binding to their seed sequences^2,5^. Given that, we identified candidate target miRNAs of the most expressed circular RNAs in gastric tissues. We realized that the seed sequence is the key that may link circRNAs, miRNAs, miRNAs target genes and circular RNAs-derived genes. Therefore, we searched for the candidate target miRNAs by identifying the miRNAs that regulates such circular RNA-derived gene and by confirming that the complementary seed sequence is present in the circRNA sequence.

**Figure 3:**
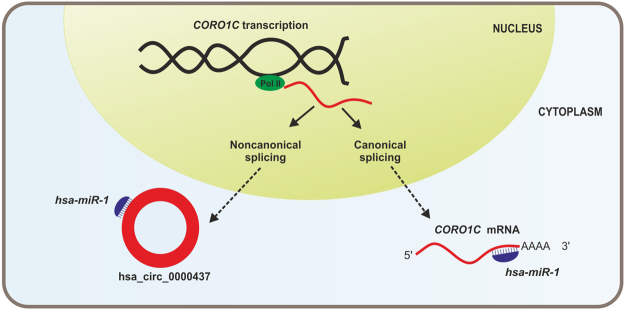
Simulation of the relation between *CORO1C*, hsa_circ_0000437 and *hsa-miR-1*. Pol II: RNA polymerase II.

After this analysis, to consolidate the candidate target miRNAs, we compared them with the differentially expressed miRNAs identified in the same samples of this study, which were obtained previously by RNA-Seq by our group [data not published]. We found five candidate miRNAs potentially regulated by five circRNAs. All of them were previously described in gastric cancer (Table 3). In Fig. 3, we illustrated the interaction between *CORO1C*, hsa_circ_0000437 and *hsa-miR-1*.

**Table 3:** Candidate target microRNAs of some of the high expressed circular RNAs in gastric tissue.

Circular RNAs		Target microRNAs		
Name	Gene	Name	Number of seed matches	Refs in gastric cancer
hsa_circ_0001340	*TMCC1*	*hsa-miR-452–5p*	1	[30,31]
hsa_circ_0000419	*RAB3IP*	*hsa-miR-145–5p*	1	[32–34]
hsa_circ_0001112	*DGKD*	*hsa-miR-375*	3	[35–37]
hsa_circ_0000284	*HIPK3*	*hsa-miR-224–5p*	1	[31,38]
hsa_circ_0000437	*CORO1C*	*hsa-miR-1*	1	[39]

Unlike CDR1as, some studies have demonstrated that most circRNAs would have only 1–2 miRNA binding sites^13,31^. Our data corroborate to these studies given that most of the circRNA identified have only one miRNA-binding site, except for hsa_circ_0001112 that have three binding sites (Table 3).

We also analyzed the distribution of the expressed circular RNAs in gastric tissue without cancer, matched tumor-adjacent gastric tissue and gastric cancer samples. The Fig. 1b shows that there are exclusive circular RNAs of each group, but also there are common circular RNAs between them. Differential expression analysis showed that of the 27 circular RNAs in common between the three groups, five are significantly different (Table 4).

**Table 4:** List describing the five differentially expressed circular RNAs in gastric tissue. The differential expression was evaluated with negative binomial regression adjusting for common and tagwise variation, and p-values were adjusted for multiple testing using a FDR procedure.

Circular RNA	Gene	Gene official name	P-value	Driver gene*
hsa_circ_0001136	*ASXL1*	Additional sex combs like 1	8,3E-04	Yes
hsa_circ_0000284	*HIPK3*	Homeodomain interacting protein kinase 3	9,0E-04	No
hsa_circ_0000211	*SFMBT2*	Scm-like with four mbt domains 2	9,8E-04	No
hsa_circ_0004771	*NRIP1*	Nuclear receptor interacting protein 1	4,6E-05	No
hsa_circ_0000524	*RBM23*	RNA binding motif protein 23	1,9E-04	No

*According to Vogelstein *et al*.^41^.

**Figure 4:**
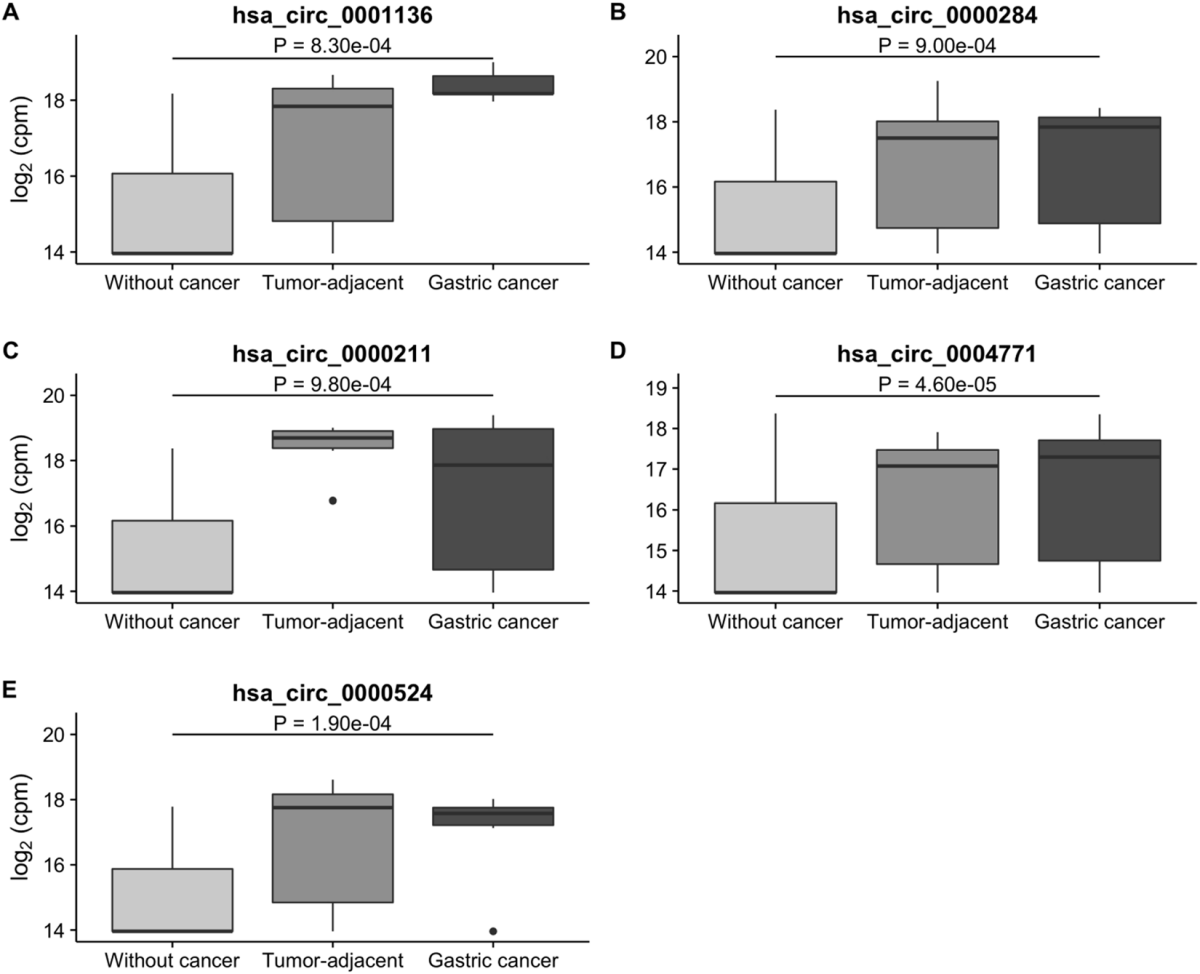
Expression of the five differentially expressed circular RNAs in gastric tissue. This analysis was performed by comparing the samples without cancer with both tumor-adjacent and gastric cancer samples combined.

The differential expression analysis was performed by comparing the samples without cancer with both tumor-adjacent and gastric cancer samples combined. All five differentially expressed circular RNAs are exonic, and were found down regulated in samples without cancer (Fig. 4).

## Discussion

Circular RNAs are a novel class of regulatory noncoding RNAs with yet unknown impact on the cellular machinery. Our study is the first to investigate and describe all circular RNAs expressed in adult human gastric tissue, comprising patients without gastric cancer, matched tumor-adjacent gastric tissue and gastric cancer samples.

We found that the matched tumor-adjacent gastric samples were the group with the highest number of circular RNAs identified, followed by gastric cancer and samples of patients without gastric cancer (Fig. 1a). Most of the previous studies about circular RNAs global expression in human cancers used only the matched tumor-adjacent samples as normal control. In all these studies, the expression of circular RNAs in cancer is down-regulated in comparison to the matched tumor-adjacent tissue^13,18,19,32^. These data suggest that the abundant expression of circular RNAs in tumor-adjacent tissue samples is a general pattern in several types of cancer, including gastric cancer.

Circular RNAs expressions were analyzed in gastric cancer in some previous studies. However, these studies used matched tumor-adjacent as control^13,33–38^. The use of adjacent tissue for comparison purposes can lead to biases since the evidences have demonstrated the field cancerization in gastric tissue surrounding the tumors^26,27^. Thus, we chose to investigate the circular RNAs expression in patients without gastric cancer, matched tumor-adjacent gastric and gastric cancer samples.

Our data suggests that circular RNAs abundance in tumor-adjacent tissue may be somehow related to gastric carcinogenesis, given its similarity to gastric cancer tissue. Most of the highest expressed circRNA genes in gastric cancer samples are also present in tumor-adjacent tissue (*CFLAR*, *CORO1C*, *HIPK3*, *ASXL1* and *SFMBT2*) (Table 2).

It is possible that the circular RNAs are not essential molecules in fully developed tumors, explaining their high expression in tumor-adjacent tissues. Bachmayr-Heyda *et al*.^18^ showed that the expression of circular RNAs in colorectal cancer cell lines is even smaller than those in colorectal cancer tissue. The cancer cell lines have a higher proliferation rate and are pure cancer cells, indicating that cancerous cells do not require a high level of circular RNAs to maintain their malignant features.

Although most circular RNAs does not have its function completely understood, it is possible to estimate their cellular role by performing a functional enrichment analysis of their derived genes. GO enrichment indicated that the gastric tissue without gastric cancer circular RNAs-derived genes were enriched for the process of bacterial invasion of epithelial cells, which is a natural process in stomach (Fig. 2). This KEGG pathway was also enriched in tumor-adjacent samples, but not in gastric cancer samples, indicating the cellular loss of function typically found in cancer.

Previous studies have discussed the potential function of circRNAs as miRNA sponges. Memczak *et al*.^5^ reported that the circRNA CDR1as (or ciRS-7) harbors about 70 binding sites for miR-7 seed. However, a deeper analysis showed that most circRNAs have less than 10 miRNA binding sites, indicating that miRNA sponging by circRNAs may not require a large number of binding sites^31^. To further investigate this information, we identified the potential circRNAs target and found five candidate miRNAs, and most of them present only one target site (Table 3).

All five candidate miRNAs were found differentially expressed between patients without gastric cancer, matched tumor-adjacent gastric and gastric cancer samples (data not shown), and previously described in association with gastric cancer in the literature. Their expressions were correlated with several features of gastric cancer, such as drug resistance, proliferation, invasion, migration and cell growth in gastric cancer^39–46^.

The Fig. 3 illustrates how complex and dynamic is the interaction between circRNA, mRNA and circRNA-derived gene. *CORO1C* gene produces circRNA and mRNA by noncanonical and canonical splicing, respectively, and both types of RNA may interact with the same miRNA. Circ-CORO1C blocks *hsa-miR-1*, while CORO1C mRNA is blocked by *hsa-miR-1*. It suggests that the circRNA production may be a gene mechanism to ensure its own mRNA translation.

Given that, regarding the type (circRNA, ciRNA or EIciRNA), circular RNAs seem to be a positive self-mechanism of gene regulation by sponging miRNAs or by interacting with RNA polymerase II.

To identify circular RNAs with potential to become gastric cancer biomarkers, we performed differential expression analysis. Among the five differentially expressed circRNAs, hsa_circ_0001136 is derived from *ASXL1*, which is a driver gene involved in chromatin modelling^47^ (Table 4).

Hsa_circ_0000284 (*HIPK3* gene) was found differentially expressed in gastric tissues (Table 4). Given that this circRNA is overexpressed in tumor-adjacent and gastric cancer samples, and also may regulate *hsa-miR-224–5p* (Table 3), the interaction between hsa_circ_0000284 and *hsa-miR-224* is possibly involved in gastric carcinogenesis. In fact, this circRNA was found overexpressed in seven types of cancer, including gastric, and related to cell proliferation^13^. *Hsa-miR-224* was also described in association to gastric cancer^45^.

Circular RNAs have some particularities that made them potential biomarkers of both physiological and pathological processes. Besides being abundant, stable and resistant, their little invasiveness remarkably increases its potential, since their expression can be accessed by body fluids^14^. Shao *et al*.^34^ demonstrated that the expression of circular RNA can be accessed by gastric juice, suggesting their potential as biomarker for disease screening.

Overall, our results revealed that the circular RNAs is overexpressed in tumor-adjacent and in gastric cancer samples in comparison to samples without cancer. We showed the presence of field cancerization in gastric cancer, indicating that the tumor-adjacent tissue cannot be considered as normal tissue. We also found five differentially expressed circRNAs that may become novel biomarkers of gastric cancer and need to be further validated. Nevertheless, our results support the hypothesis of circular RNAs representing a novel factor in the dynamic epigenetic network of gene regulation, which involves the miRNAs and its mRNAs targets and the circular RNAs-derived genes. Further studies are needed to elucidate the roles and the functional relevance of the circular RNAs in human diseases.

## Methods

### Clinical samples

We included tissue samples of patients without gastric cancer (n = 8), gastric cancer (n = 8) and matched tumor-adjacent (n = 8), from the Universitary Hospital of João de Barros Barreto of the Federal University of Pará. All samples were collected, stored in *RNAlater* (Thermo Fisher Scientific) and frozen in liquid nitrogen until RNA total isolation. The study including all experimental protocols was approved by the *Ethics Committee of the Center of Oncology Research of the Federal University of Pará* (No. 1.432.512). All study participants or their legal guardian provided informed written consent in accordance with the Helsinki Declaration. The methods were performed in accordance to the approved guidelines.

### RNA isolation

Total RNA was isolated from tissue samples by using TRIzol Reagent (Thermo Fisher Scientific) following the manufacture’s protocol. Total RNA integrity and amount were evaluated by Qubit 2.0 Fluorometer (Thermo Fisher Scientific), NanoDrop ND-1000 (Thermo Fisher Scientific) and 2200 Tape Station System (Agilent). The integrity criteria were values between 1.8 and 2.2 (A _260/280_), >1.8 (A_260/230_), and RIN ≥ 5.

### Circle-Seq sample treatment, library synthesis, sequencing and analysis

First, a step of circular RNA enrichment was made by treating the total RNA with 3U of RNase R (Epicentre), followed by 15 minutes at 37 °C. After this, the treated RNA was re-quantified, and 1 μg of treated RNA per sample was used as input to prepare the libraries. We synthesized 24 libraries by using TruSeq Stranded Total RNA Library Prep with Ribo-Zero Gold (Illumina), which already has a step of rRNA depletion included. The libraries quality was controlled with 2200 TapeStation (Agilent), normalized to 10 nM and sequenced on a MiSeq Sequencing System (Illumina) by using the MiSeq Reagent Kit v3 (Illumina).

FASTQ was trimmed, cropped and adapters contaminant were removed (Trimmomatic v.0.36). The resulting reads were aligned to human genome (hg19) using both BWA (v.0.7) and STAR (v.2.5), which were processed by CIRI (v.2.0)^48^ and CIRCexplorer2 (v.2.2)^49^, respectively, to detect head-to-tail back-spliced junctions. We considered only the junctions detected by both tools to improve prediction accuracy^50^.

The detected circRNA list was used to made a Venn diagram (Venny 2.1 - http://bioinfogp.cnb.csic.es/tools/venny/index.html) representing the distribution of the expressed circular RNAs among gastric tissue without gastric cancer, gastric cancer samples and matched tumor-adjacent samples. All other graphics and statistical analyses were performed by using R (v.3.3). The read count was normalized and compared between groups using edgeR (v.3.18) package (REF).

### Circular RNAs functional analysis

Gastric circular RNAs-derived genes were selected to perform for the functional enrichment analysis. This analysis was performed by DAVID Bioinformatics Resources v6.8 (https://david.ncifcrf.gov). All enriched KEGG pathways were plotted. P-values were adjusted by using Bonferroni’s correction.

### Selection of the candidate target microRNAs

The candidate target miRNAs were predicted by searching which miRNA has the circular RNA-derived gene as a target. This search was performed by using the miRTarBase, an experimentally validated microRNA-target interactions database (http://mirtarbase.mbc.nctu.edu.tw). After that, we searched for a complementary region to miRNA seed sequence in circular RNA, and confirmed that the predicted miRNA was found differentially expressed in gastric cancer [data not published].

## Electronic supplementary material

Supplementary material

## References

[1] Jeck WR (2013). Circular RNAs are abundant, conserved, and associated with ALU repeats. RNA.

[2] Hansen T (2013). Natural RNA circles function as efficient microRNA sponges. Nature.

[3] Sanger K, Riesner G (1976). & Kleinschmidt. Viroids are single-stranded covalently closed circular RNA molecules existing as highly base-paired rod-like structures. Proceedings of the National Academy of Sciences.

[4] Cocquerelle C, Daubersies P, Majerus MA (1992). Splicing with inverted order of exons occurs proximal to large introns. The EMBO Journal.

[5] Memczak S (2013). Circular RNAs are a large class of animal RNAs with regulatory potency. Nature.

[6] Rybak-Wolf, A. *et al*. Circular RNAs in the Mammalian Brain Are Highly Abundant, Conserved, and Dynamically Expressed. *Molecular Cell*. **58**, 10.1016/j.molcel.2015.03.027 (2015). 10.1016/j.molcel.2015.03.027 25921068

[7] Guo J, Agarwal V, Guo H, Bartel D (2014). Expanded identification and characterization of mammalian circular RNAs. Genome Biology.

[8] Zhang, Y. *et al*. Circular Intronic Long Noncoding RNAs. *Molecular Cell* **51**, (2013). 10.1016/j.molcel.2013.08.017 24035497

[9] Salzman J, Chen R, Olsen M, Wang P, Brown P (2013). Cell-Type Specific Features of Circular RNA Expression. PLoS Genetics.

[10] Kelly, S., Greenman, C., Cook, P. & Papantonis, A. Exon Skipping Is Correlated with Exon Circularization. *Journal of Molecular Biology* **427**, 10.1016/j.jmb.2015.02.018 (2015). 10.1016/j.jmb.2015.02.018 25728652

[11] Ashwal-Fluss, R. *et al*. circRNA Biogenesis Competes with Pre-mRNA Splicing. *Molecular Cell* **56**, 10.1016/j.molcel.2014.08.019 (2014). 10.1016/j.molcel.2014.08.019 25242144

[12] Capel B, Swain A, Nicolis S, Hacker A, Walter M (1993). Circular transcripts of the testis-determining gene Sry in adult mouse testis. Cell.

[13] Zheng Q (2016). Circular RNA profiling reveals an abundant circHIPK3 that regulates cell growth by sponging multiple miRNAs. Nat Commun.

[14] Vidal A, Sandoval G, Magalhães L, Santos S, Ribeiro-dos-Santos Â (2016). Circular RNAs as a new field in gene regulation and their implications in translational research. Epigenomics.

[15] Lukiw W (2013). CircularRNA (circRNA) in Alzheimer’s disease (AD). Frontiers in Genetics.

[16] Burd CE (2010). Expression of linear and novel circular forms of an INK4/ARF-associated non-coding RNA correlates with atherosclerosis risk. PLoS Genet.

[17] Xu H, Guo S, Li W, Yu P (2015). The circular RNA Cdr1as, via miR-7 and its targets, regulates insulin transcription and secretion in islet cells. Sci Reports.

[18] Bachmayr-Heyda A (2015). Correlation of circular RNA abundance with proliferation - exemplified with colorectal and ovarian cancer, idiopathic lung fibrosis, and normal human tissues. Scientific Reports.

[19] Xuan L (2016). Circular RNA: a novel biomarker for progressive laryngeal cancer. Am J Transl Res.

[20] Qin M (2016). Hsa_circ_0001649: A circular RNA and potential novel biomarker for hepatocellular carcinoma. Cancer Biomark.

[21] Sand M (2016). Circular RNA expression in cutaneous squamous cell carcinoma. J Dermatol Sci.

[22] Chiurillo M (2015). Role of the Wnt/β-catenin pathway in gastric cancer: An in-depth literature review. World J Exp Medicine.

[23] Slaughter DP, Southwick HW, Smejkal W (1953). Field cancerization in oral stratified squamous epithelium; clinical implications of multicentric origin. Cancer.

[24] Braakhuis BJ, Tabor MP, Kummer JA, Leemans CR, Brakenhoff RH (2003). A genetic explanation of Slaughter’s concept of field cancerization: evidence and clinical implications. Cancer Res..

[25] Chai H, Brown RE (2009). Field effect in cancer-an update. Ann. Clin. Lab. Sci..

[26] Endoh M (2005). RASSF2, a potential tumour suppressor, is silenced by CpG island hypermethylation in gastric cancer. Br. J. Cancer.

[27] Assumpcao P (2015). High-Throughput miRNA Sequencing Reveals a Field Effect in Gastric Cancer and Suggests an Epigenetic Network Mechanism. Bioinformatics and Biology Insights.

[28] Hammond S (2015). An overview of microRNAs. Advanced Drug Delivery Reviews.

[29] Zhang X (2014). Complementary Sequence-Mediated Exon Circularization. Cell.

[30] Kanehisa M, Furumichi M, Tanabe M, Sato Y, Morishima K (2017). KEGG: new perspectives on genomes, pathways, diseases and drugs. Nucleic Acids Res.

[31] Jeck WR, Sharpless NE (2014). Detecting and characterizing circular RNAs. Nat. Biotechnol..

[32] Shang X (2016). Comprehensive Circular RNA Profiling Reveals That hsa_circ_0005075, a New Circular RNA Biomarker, Is Involved in Hepatocellular Crcinoma Development. Medicine.

[33] Li P (2015). Using circular RNA as a novel type of biomarker in the screening of gastric cancer. Clin. Chim. Acta.

[34] Shao Y (2017). Global circular RNA expression profile of human gastric cancer and its clinical significance. Cancer Medicine.

[35] Tian, M., Chen, R., Li, T. & Xiao, B. Reduced expression of circRNA hsa_circ_0003159 in gastric cancer and its clinical significance. *J Clin Lab Anal* e22281, 10.1002/jcla.22281 (2017). 10.1002/jcla.22281 PMC6817154 28618205

[36] Shao Y (2017). Decreased expression of hsa_circ_0001895 in human gastric cancer and its clinical significances. Tumor Biol.

[37] Huang Y, Jie N, Zou K, Weng Y (2017). Expression profile of circular RNAs in human gastric cancer tissues. Mol Med Rep.

[38] Chen J (2017). Circular RNA profile identifies circPVT1 as a proliferative factor and prognostic marker in gastric cancer. Cancer Lett.

[39] Ueda T (2010). Relation between microRNA expression and progression and prognosis of gastric cancer: a microRNA expression analysis. Lancet Oncol..

[40] Chang S (2015). miR-145 mediates the antiproliferative and gene regulatory effects of vitamin D3 by directly targeting E2F3 in gastric cancer cells. Oncotarget.

[41] Chen J (2015). Reverse Correlation between MicroRNA-145 and FSCN1 Affecting Gastric Cancer Migration and Invasion. Plos One.

[42] Tao H-Q (2016). MicroRNA-145-5p inhibits gastric cancer invasiveness through targeting N-cadherin and ZEB2 to suppress epithelial–mesenchymal transition. Oncotargets Ther.

[43] Lian S (2016). MicroRNA-375 Functions as a Tumor-Suppressor Gene in Gastric Cancer by Targeting Recepteur d’Origine Nantais. Int J Mol Sci.

[44] Tsukamoto Y (2010). MicroRNA-375 is downregulated in gastric carcinomas and regulates cell survival by targeting PDK1 and 14-3-3zeta. Cancer research.

[45] He C, Wang L, Zhang J, Xu H (2017). Hypoxia-inducible microRNA-224 promotes the cell growth, migration and invasion by directly targeting RASSF8 in gastric cancer. Mol. Cancer.

[46] Han C (2015). MicroRNA-1 (miR-1) inhibits gastric cancer cell proliferation and migration by targeting MET. Tumor Biol.

[47] Vogelstein B (2013). Cancer Genome Landscapes. Science.

[48] Gao Y, Wang J, Zhao F (2015). CIRI: an efficient and unbiased algorithm for *de novo* circular RNA identification. Genome Biology.

[49] Zhang X-O (2016). Diverse alternative back-splicing and alternative splicing landscape of circular RNAs. Biotechfor.

[50] Hansen T, Venø M, Damgaard C, Kjems J (2016). Comparison of circular RNA prediction tools. Nucleic Acids Res.

