# Supplementary material for: The comprehensive expression analysis of circular RNAs in gastric cancer and its association with field cancerization
Source: Sci Rep. 2017 Nov 6;7:14551. doi: 10.1038/s41598-017-15061-w (PMC5673933; doi:10.1038/s41598-017-15061-w)
Supplement: Supplementary file 1 — Supplementary material [file 41598_2017_15061_MOESM1_ESM.pdf]

The comprehensive expression analysis of circular RNAs in gastric cancer and its association with field cancerization

Amanda Ferreira Vidal[1§], André M. Ribeiro-dos-Santos[1§], Tatiana Vinasco-Sandoval[1], Leandro Magalhães[1], Pablo Pinto[1], Ana KM Anaissi[2], Samia Demachki[2], Paulo Pimentel de Assumpção[2], Sidney Emanuel Batista dos Santos[1,2], Ândrea Ribeiro-dos-Santos[1,2]

[1] Laboratory of Human and Medical Genetics, Federal University of Pará, Belém, Pará, Brazil.

[2] Center of Oncology Research, Federal University of Pará, Belém, Pará, Brazil.

[§]These authors contributed equally to this work.

Correspondence and requests for materials should be addressed to Â. Ribeiro-dos-Santos. (akelyufpa@gmail.com)

Supplementary information

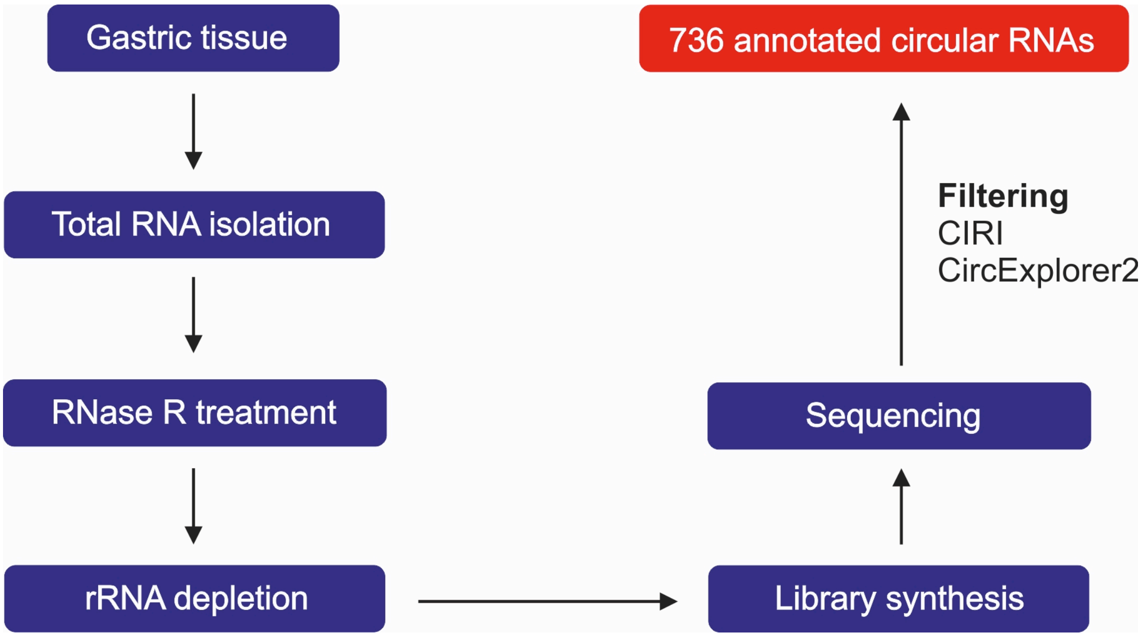

Supplementary Fig. 1 | Circular RNAs detection workflow.

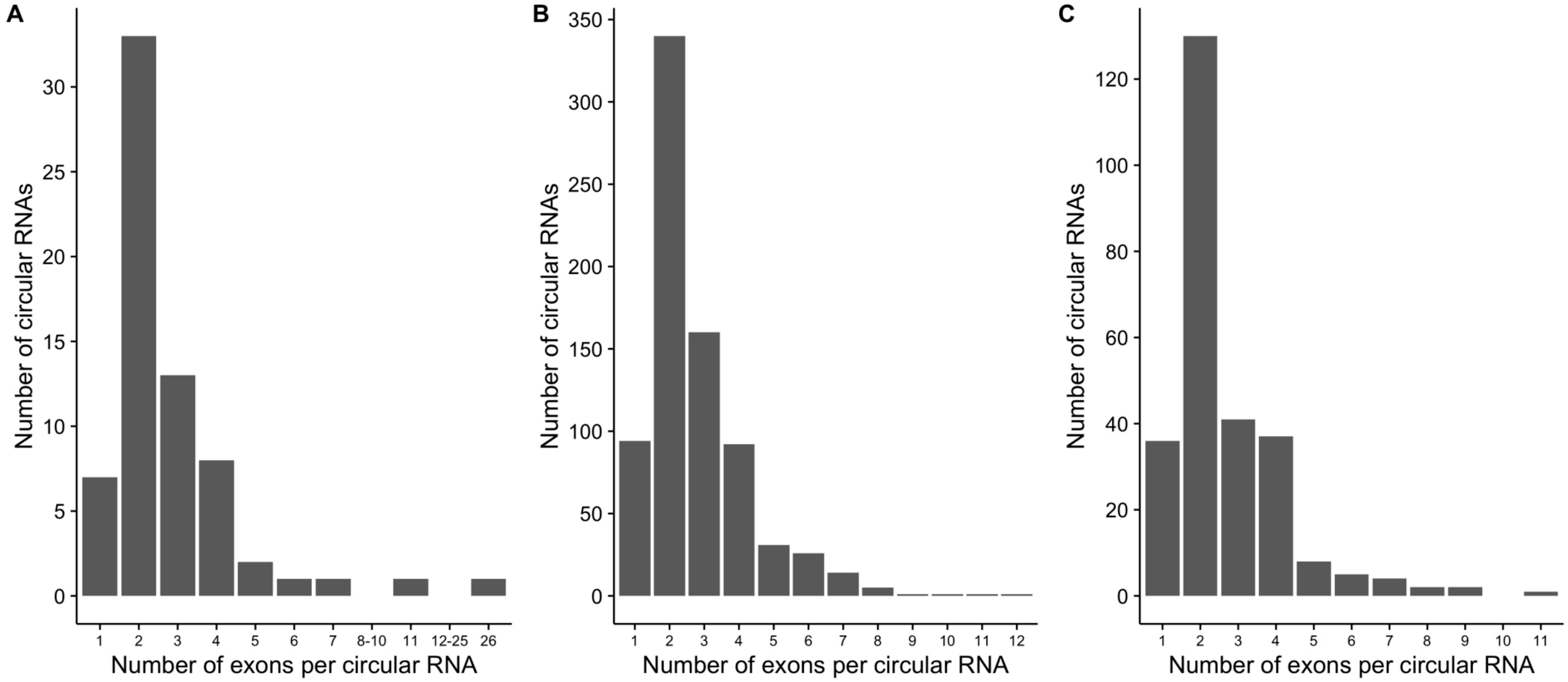

Supplementary Fig. 2 | Number of exons per annotated circular RNAs in gastric tissue. (A) Gastric tissue without gastric cancer. (B) Matched tumor-adjacent gastric tissue. (C) Gastric cancer.

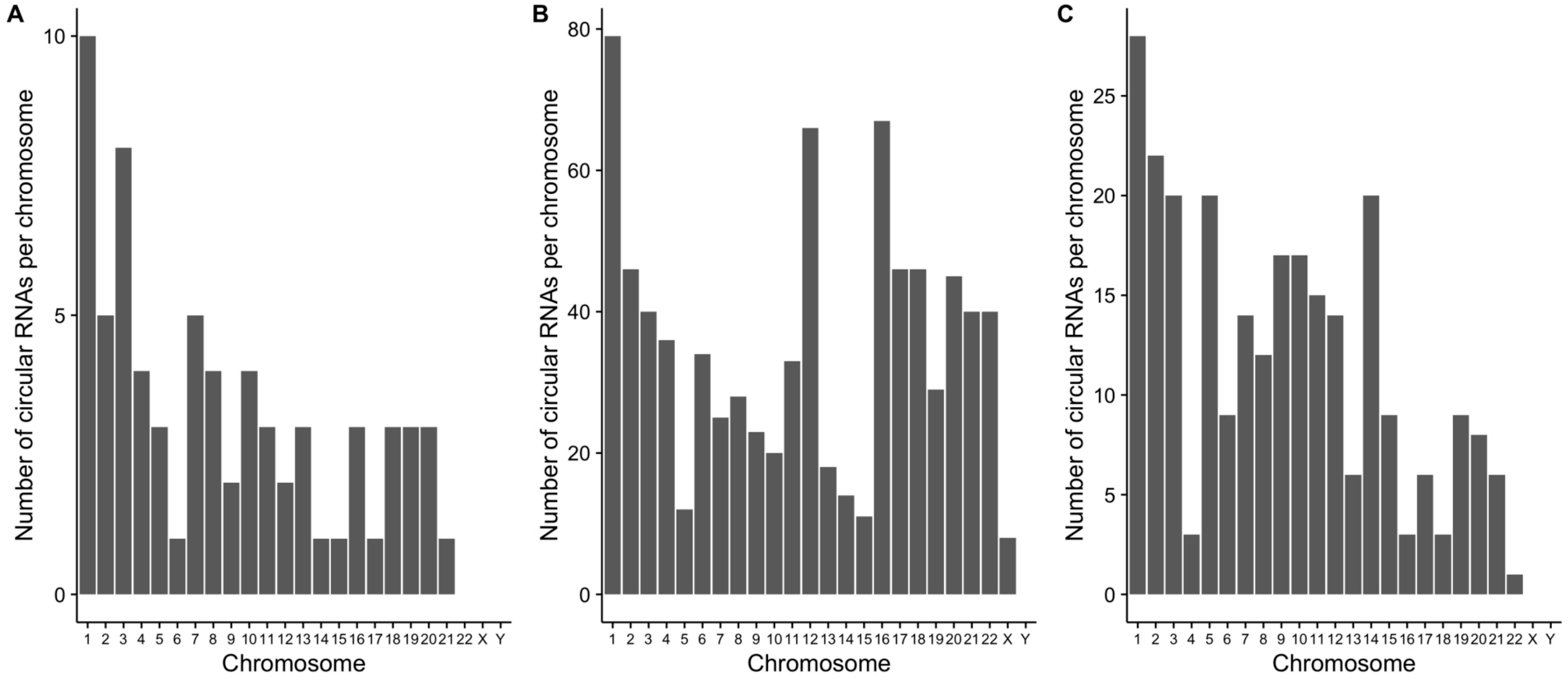

Supplementary Fig. 3 | Number of circular RNAs per human chromosome. (A) Gastric tissue without gastric cancer. (B) Matched tumor-adjacent gastric tissue. (C) Gastric cancer.

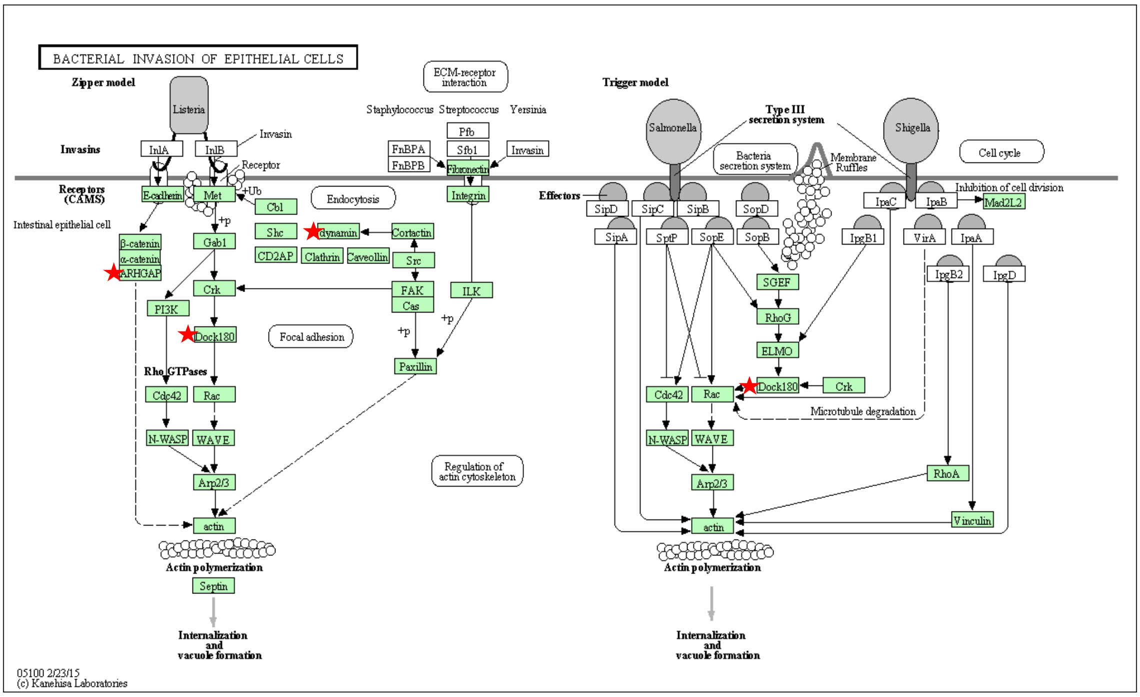

Supplementary Fig. 4 | Scheme of the KEGG pathway of bacterial invasion of epithelial cells[30]**.** This pathway was enriched in gastric tissue without gastric cancer and in matched adjacent tumor gastric tissue.
